# Robot-assisted T12 pediculectomy and resection of a ventral thoracic meningioma

**DOI:** 10.3171/2023.7.FOCVID2372

**Published:** 2023-10-01

**Authors:** Mahmudur Rahman, Huy Truong, Aditya Vedantam

**Affiliations:** Department of Neurosurgery, Medical College of Wisconsin, Milwaukee, Wisconsin

**Keywords:** intradural extramedullary lesion, intradural spinal tumor, spinal meningioma, thoracic meningioma, transpedicular approach

## Abstract

Spinal meningiomas represent 25%–45% of intradural spinal tumors and are commonly seen in the thoracic spine. Ventral midline spinal meningiomas in the thoracic spine are challenging lesions to resect given their location in relation to the spinal cord. Resection for symptomatic or growing lesions requires adequate bone removal to limit retraction of the spinal cord. Surgical adjuncts such as intraoperative navigation, robotics, and ultrasound can improve the efficiency of and safety for resection of these lesions. The authors present a case of a complete resection of a ventral thoracic meningioma using a T12 transpedicular approach with robot-assisted navigated pediculectomy and intraoperative ultrasonography.

## Transcript

The authors present a case demonstrating complete resection of a ventral thoracic meningioma^1–6^ using a T12 transpedicular approach with robot-assisted and navigated pediculectomy and intraoperative ultrasonography.^1,2^

### 0:36 Patient Presentation.

A 61-year-old female presented with an intradural ventral thoracic mass. She had intermittent low-back pain with mild gait imbalance. She had no weakness or numbness in her lower limbs or new bowel or bladder dysfunction. Physical examination demonstrated full strength in upper and lower extremities except for right ankle, which had limited movement due to prior foot surgery. Patient had intact sensation throughout. She did not exhibit hyperreflexia or clonus in her lower extremities.

### 1:12 Preoperative Imaging.

Preoperative sagittal T2-weighted MRI showed a ventral T2 isointense intradural extramedullary lesion with cord compression at T12 as well as faint intramedullary T2 hyperintensity. The axial T2 image showed that the spinal cord was rotated toward the left and the lesion was in the midline but eccentric to the right side.

Sagittal and axial contrasted MRI of the thoracic spine showed homogeneous enhancement within the ventral intradural extramedullary T12 lesion.

Noncontrasted CT scan of the thoracic spine showed that the ventral T12 lesion was calcified. Imaging of the rest of her neuraxis did not show any additional lesions.

### 2:05 Surgical Plan.

Given the degree of cord compression, intramedullary signal change, and symptoms of mild thoracic myelopathy, we recommended surgical resection of this lesion. The surgical plan was a T11–12 laminectomy with a right T12 robot-assisted pediculectomy and resection of ventral intradural extramedullary mass. Given that the calcified tumor was visualized on the preoperative navigation CT, the surgical robotic system was used for presurgical planning as well as to perform the pediculectomy. We also performed a T10–L2 robot-assisted pedicle screw fixation and posterolateral fusion. We elected to perform fusion to prevent postsurgical thoracolumbar instability. We also planned to utilize intraoperative ultrasound, neuromonitoring, and microscope for microsurgical dissection.

### 3:00 Initial Operative Steps and Pediculectomy.

The patient was placed in a prone position on a Jackson table. Fluoroscopy was used to identify the T12 pedicle and a midline incision from T10 to L2 was made. Next, a subperiosteal dissection was performed, and a clamp was placed on the transverse process of T12. A right iliac crest pin was then used to anchor the surgical robot, and fluoroscopy was used to register the presurgical CT to the patient’s anatomy.

This presurgical planning CT shows the screw trajectories for the T10–L2 fusion.

The calcified ventral lesion is visible on the axial image at T12. The right T12 screw was planned to visualize the trajectory of the pediculectomy that needed to be performed at that level. Using the robotic navigation and arm, the pedicle was drilled and tapped up to the posterior edge of the vertebral body. This facilitated a more efficient and safer pediculectomy to access the tumor.^7^

This image shows the results of pediculectomy.

### 4:11 Intraoperative Ultrasound.

Laminectomy with facetectomy and pediculectomy was performed to access the tumor. Intraoperative ultrasound confirmed adequate bone removal, and the entire tumor was visualized. This ultrasound image is showing the hyperechoic rim of the ventral intradural extramedullary lesion with the spinal cord draped over it.

### 4:21 Dura Incision.

The dura is being opened off midline to the right. The arachnoid is being opened separately and tacked up with vascular mini clips.

### 4:57 Tumor Exposure and Resection.

The partially calcified tumor is visible displacing the cord dorsally and the nerve roots laterally. The roots around the tumor are being moved to develop the plane between tumor and spinal cord. The dentate ligament is being sharply dissected to prevent traction on the spinal cord during surgery. The superior and inferior poles of the tumor are being identified.

The tumor capsule is being opened. At this stage, specimens were sent for permanent pathology. The tumor is being internally debulked using the ultrasonic aspirator.

Arachnoid adhesions at the poles were cut to mobilize the tumor. Ventral dural attachment was sharply dissected. All visible tumor was resected, and the ventral dura was coagulated. Ultrasound was performed in addition to visual inspection and did not reveal any residual lesion There was no change in the baseline SSEPs or MEPs throughout the case.

### 7:25 Dural Repair.

Dura was repaired with 5-0 Prolene in a watertight fashion. The wound was irrigated with antibiotic irrigation. At this point, we performed an ultrasound once more. This ultrasound image shows no signs of residual tumor or intradural hematoma. After this, the facet joints and transverse processes were decorticated. Autograft and morselized allograft was placed for posterolateral arthrodesis. The wound was then closed in layers.

### 8:21 Postoperative Imaging.

The patient had no new neurological deficits after surgery. She experienced a transient postoperative ileus that resolved prior to discharge home. At her last follow-up visit approximately 6 months after surgery, she reported no new neurological deficits and improving presurgical symptoms. Postoperative T2-weighted and contrasted T1-weighted MRIs showing improved position of the spinal cord within the canal and no residual tumor.

CT scan of the thoracolumbar spine shows the laminectomy defect and removal of the ventral calcified lesion as well as evidence of the right T12 pediculectomy.

Postoperative thoracolumbar x-ray shows the T10–L2 fusion hardware to be in stable position.

Final pathology showed that the lesion was a WHO grade 1 meningioma with abundant calcified psammoma bodies and extremely rare mitotic figures.

## Disclosures

The authors report no conflict of interest concerning the materials or methods used in this study or the findings specified in this publication.

## Author Contributions

Primary surgeon: Vedantam. Assistant surgeon: Truong. Editing and drafting the video and abstract: Vedantam, Rahman. Critically revising the work: Vedantam, Rahman. Reviewed submitted version of the work: all authors. Approved the final version of the work on behalf of all authors: Vedantam. Supervision: Vedantam.

## Supplemental Information

### Patient Informed Consent

The necessary patient informed consent was obtained in this study.
